# Antidepressant prescribing in Australian primary care: time to reevaluate

**DOI:** 10.5694/mja2.52645

**Published:** 2025-04-02

**Authors:** Katharine A Wallis, Anna King, Joanna Moncrieff

**Affiliations:** ^1^ University of Queensland Brisbane QLD; ^2^ University College London London United Kingdom

**Keywords:** Antidepressive agents, Prescribing, Guidelines as topic

Around one in seven Australians is now taking antidepressants (3.9 million people, 14%),^1^ and the prevalence of use is rising.^2^, ^3^ Two antidepressants, sertraline and escitalopram, are now in the top ten drugs by defined daily dose per 1000 population per day.^4^ In Australia, women are prescribed antidepressants at 1.5 times the rate of men, and older people (aged ≥ 65 years) are twice as likely to be prescribed antidepressants as younger people (aged < 65 years).^2^, ^5^ Around 26% of people aged 75 years or older are taking antidepressants.^2^ Most psychological distress or mental illness is managed in primary care, and antidepressant prescribing is overwhelmingly in primary care, with general practitioners prescribing 92% of antidepressants in Australia.^1^

The explanation for higher antidepressant prescribing in women and older people remains uncertain. Social issues such as loneliness, grief or abuse are not resolved by antidepressants. Leading psychiatrists argue that we cannot exclude the rising prevalence of depression or “better recognition (by both patients and clinicians) and treatment of depression in primary care” as explaining the increase in antidepressant use.^6^ Yet, there is evidence from primary care that people are prescribed antidepressants when clinical guideline criteria are not met, more commonly women.^7^ Further, it is unlikely that 14% of the Australian population would fit the clinical guideline criteria for antidepressant use. Clinical guidelines recommend non‐drug interventions as first line for anxiety and less severe depression, and “in severe major depression” antidepressant therapy for “6 to 12 months, then consider deprescribing”.^8^ It is widely accepted that antidepressant effects “may be minimal or non‐existent, on average, in patients with mild or moderate symptoms”.^9^ A recent systematic review and network meta‐analysis “found some forms of exercise to have stronger effects than [selective serotonin reuptake inhibitors] alone”.^10^ Debate about antidepressants and suicide is ongoing, but the United States Food and Drug Administration has long directed manufacturers to include a boxed warning for increased suicide risk with antidepressants in younger people.^11^

Regardless of whether initiation was justified or not, evidence suggests that the increase in antidepressant use is due largely to rising long term use (longer than 12 months); that is, people failing to stop antidepressants.^12^, ^13^ Around half of people taking antidepressants take them for longer than two years.^14^ In the ongoing RELEASE (Redressing Long‐Term Antidepressant Use) trial in 26 general practice clinics in southeast Queensland, we identified 12 069 adults (aged ≥ 18 years) prescribed at least one of 15 commonly used antidepressants (tricyclic antidepressants excluded), of whom 6081 (50%) were long term users.^15^

The justification for long term antidepressant prescribing is unclear. There is little evidence showing benefit in long term antidepressant therapy, although there is evidence of harm. Adverse effects include lethargy or fatigue, weight gain, emotional numbing, and sexual dysfunction that may be permanent.^16^ In older age, antidepressants are associated with increased risk of falls and fracture, hyponatraemia, seizures, admission to residential care and all‐cause mortality.^17^, ^18^ Long term antidepressant therapy has been justified by results of relapse prevention trials showing higher rates of relapse among individuals randomly assigned to discontinue antidepressants compared with those who continue them.^19^ Yet, arguably these trials stop antidepressants too precipitously and misinterpret physiological withdrawal symptoms as relapse.^20^ Antidepressant withdrawal symptoms include emotional symptoms such as anxiety, irritability, low mood and tearfulness,^21^ making the confusion or misdiagnosis easy to understand. These symptoms have been measured in clinical trials as “depression” and “anxiety” using scales such as the Patient Health Questionnaire‐9 (PHQ‐9) and General Anxiety Disorder‐7 (GAD‐7).^21^ Misdiagnosis of withdrawal symptoms can lead to mismanagement with reinstating or switching and continuing antidepressants, sometimes long term.^22^

Although disagreement continues about the incidence of withdrawal symptoms, there is a growing body of evidence that antidepressant withdrawal symptoms are common and can be severe and long‐lasting, especially with longer term use.^23^, ^24^ Thousands of people who have experienced these symptoms gather in online support groups and report life‐changing consequences or being unable to stop taking antidepressants despite their desire to do so.^21^ Consumer medicine information leaflets list symptoms that may occur after stopping antidepressants to include “agitation, nervousness, anxiety”, and include the warning “Do not let yourself run out of tablets over the weekend”.^25^ This warning likely relates to the risk of withdrawal symptoms (“agitation, nervousness, anxiety”), since relapse would take longer to manifest. Even if only 2% of users experience severe withdrawal symptoms, the scale of the problem is alarming given that nearly 2 million Australians are long term antidepressant users.

An article published in 2024 argued that it is the patient's preference to continue antidepressants.^6^ However, lack of awareness and recognition of withdrawal symptoms,^22^ and their potential severity, means that people may not be able to make informed and free choices.

Internationally, rising antidepressant use and a growing appreciation of the difficulties that some people experience stopping antidepressants have led to calls for changes in prescribing^26^ and to the publication of guidance for stopping antidepressants via slow hyperbolic tapering of drug dose.^27^, ^28^, ^29^ The Royal Australian College of General Practitioners has officially recognised the *Maudsley deprescribing guidelines* (which recommend hyperbolic tapering) as an Accepted Clinical Resource,^29^, ^30^ but otherwise guidance in Australia for how to stop antidepressants remains unchanged and unhelpful.^31^, ^32^ Further, some psychiatrists continue to argue against antidepressant deprescribing,^6^ citing as evidence the Royal Australian and New Zealand College of Psychiatrists’ (RANZCP) guidelines, which claim that hyperbolic tapering is impractical and not feasible, without suggesting any viable alternative.^32^ The lack of diversity in the RANZCP guidelines panel, which was overwhelmingly male, is relevant, noting that women are prescribed antidepressants at a higher rate than men and that guideline authors should represent consumer demographics. As highlighted in an editorial, to some extent “recommendations are guided by the opinions, clinical experience, and composition of the guideline development panel. It is consequently vital that these panels represent the community to whom the guidelines will apply”.^33^ Guideline panels should also include representation from the community for whom the guidelines are written, that is, mostly general practitioners, since general practitioners and psychiatrists manage a different spectrum of mental illness.

How best to minimise withdrawal symptoms to enable people to successfully stop antidepressants requires further research. Although slow hyperbolic tapering of drug dose can help to minimise withdrawal symptoms, we do not yet know the optimal tapering regimen. Nor do we know that this approach will work in everyone. It is possible that some people will not be able to stop their antidepressant due to the severity of withdrawal symptoms.^21^ If so, then informed consent discussions about starting antidepressants need to include information that, once started, it may not be possible to stop antidepressants, and patients may have to take them for the rest of their life with associated risks. For most people there are better alternatives available.^26^

The evidence suggests that antidepressants are overprescribed in Australia: patients, more commonly women and older people, are both started on antidepressants when clinical guideline criteria are not met,^7^ and continued on antidepressants for longer than clinical guidelines recommend for most people,^12^ where the potential harms likely outweigh the potential benefits, and where better alternatives are available.^26^

To improve the health and wellbeing of Australians, it is timely that we reevaluate antidepressant prescribing in primary care. Given the sheer number of people now taking antidepressants long term and the difficulty that some people face stopping antidepressants, there needs to be better recognition (by both patients and practitioners) and management of antidepressant withdrawal symptoms and support for people to safely stop.^34^ This includes practical resources for use in clinical practice, such as tapering plans with step‐by‐step instructions for slowly decreasing drug dose and advice for accessing the mini doses used in tapering. The need for such resources is reflected in the unprecedented demand we have received for the resources being tested in the RELEASE trial (now recognised as an Accepted Clinical Resource by the Royal Australian College of General Practitioners),^15^, ^35^, ^36^ including hundreds of emails from both practitioners (general practitioners, psychiatrists, pharmacists and psychologists) and patients from across Australia and internationally. Also, given the limited efficacy of antidepressants in mild to moderate mental illness, the risk of adverse effects and the difficulties that people can experience stopping antidepressants, there needs to be better decision support and informed consent for starting antidepressants.^26^

## Open access

Open access publishing facilitated by The University of Queensland, as part of the Wiley ‐ The University of Queensland agreement via the Council of Australian University Librarians.

## Competing interests

Katharine Wallis received funding from the Australian Medical Research Future Fund (MRFF) and the National Health and Medical Research Council (NHMRC) to lead the RELEASE (Redressing Long‐term Antidepressant Use) trial. Anna King is a member of the MRFF and NHMRC funded RELEASE trial Lived Experience Advisory Group. Joanna Moncrieff is a co‐investigator on the MRFF and NHMRC funded RELEASE trial, she has been a co‐investigator on a study of antidepressant discontinuation funded by the United Kingdom's National Institute for Health and care Research, she receives modest royalties from books about psychiatric drugs, and she is co‐chair of the Critical Psychiatry Network.

## Provenance

Not commissioned; externally peer reviewed.
